# Low-Energy Collisions of Protonated Enantiopure Amino Acids with Chiral Target Gases

**DOI:** 10.1007/s13361-017-1796-7

**Published:** 2017-09-21

**Authors:** K. Kulyk, O. Rebrov, M. Ryding, R. D. Thomas, E. Uggerud, M. Larsson

**Affiliations:** 1 Department of Physics, Stockholm University, SE-10691 Stockholm, Sweden; 2 SCA R&D Centre, Sidsjövägen 2, SE-85121 Sundsvall, Sweden; 3 Department of Chemistry, University of Oslo, NO-0315 Oslo, Norway

**Keywords:** Collision induced dissociation, Chiral target gases, Chirality, Low-energy collisions, Enantiopure amino acids, Gas-phase complexes

## Abstract

Here we report on the gas-phase interactions between protonated enantiopure amino acids (l- and d-enantiomers of Met, Phe, and Trp) and chiral target gases [(*R*)- and (*S*)-2-butanol, and (*S*)-1-phenylethanol] in 0.1–10.0 eV low-energy collisions. Two major processes are seen to occur over this collision energy regime, collision-induced dissociation and ion-molecule complex formation. Both processes were found to be independent of the stereo-chemical composition of the interacting ions and targets. These data shed light on the currently debated mechanisms of gas-phase chiral selectivity by demonstrating the inapplicability of the three-point model to these interactions, at least under single collision conditions.

**Electronic supplementary material:**

The online version of this article (10.1007/s13361-017-1796-7) contains supplementary material, which is available to authorized users.

**Figure:**
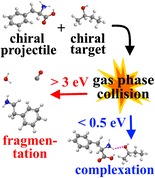
Graphical Abstract

## Introduction

Previous studies on gas-phase chiral recognition have mostly been confined to mass spectrometry-based approaches, and numerous papers have been published that have demonstrated their analytical feasibility. Four successive reviews comprehensively describe the majority of the research performed in this field up to 2017 [1–4]. The general conclusion arising from much of this work is that mass spectrometry (MS) can be used successfully for qualitative and quantitative discrimination between enantiomers. Current chiral MS-based identification methodologies can be grouped into three main classes depending on the utilized chiral recognition mechanism: host–guest diastereomeric adduct formation, guest exchange ion–molecule reactions, and collision-induced dissociation (CID) of diastereomeric complex ions [1–4].

It is evident that all the above mentioned gas-phase methods for chiral recognition are indirect probes, and, in this respect, have inherent limitations. For example, the application of these methods require standard enantiopure reference compounds and cumbersome calibration procedures. Furthermore, some of the methods require non-standard instrument modifications and theoretical modeling of the ion fragmentation patterns. It should also be mentioned that successful MS-based chiral analysis is still hampered by limited generality in that enantiomeric selectivity is often compound-dependent.

Unlike the indirect MS-based methods just described, ion mobility spectrometry (IMS), an emerging technique for gas-phase chiral separation, was proposed for direct separation of enantiomers already in the early 2000s [5]. IMS is an analytical method for gas-phase separation and identification of ions based on measurement of their mobility in a flowing buffer gas. Karas [5] patented the idea of seeding a volatile chiral dopant to the buffer gas for separating ionic enantiomers. Enantioselective clustering of dopant molecules onto the analyte ions of different chirality changes their drift times, and enables chiral recognition [5]. Crude separation of fluoxetine enantiomers using chiral 2-butanol was demonstrated in the patent [5]. Subsequently, in 2006, the same principle was employed by Dwivedi et al. [6]. Analyte enantiomers of l/d amino acids, α-methyl-l/d-glucopyranosides, and a few pharmaceutical drugs were separated by doping the nitrogen buffer gas with a volatile chiral modifier, 2-butanol. It was shown that enantioselective interactions occur between the analyte enantiomers and the chiral modifier, and that switching from (*S*)-2-butanol to (*R*)-2-butanol as the modifier resulted in the reversed enantiomer retention in the drift cell. Owing to the speed and simplicity demonstrated in these two studies [5, 6], chiral IMS became to be considered as a promising technique in gas-phase chiral analysis [1–3].

Surprisingly, in the fifteen years since the initial report [5, 6], the mechanism of enantioselective interaction behind chiral IMS is still not fully understood. Moreover, only a few successful studies utilizing the approach of doping the drift gas by a chiral modifier have been reported since these initial reports [7, 8].

Our initial interest in this area was to investigate the possibility of enantioselective interactions in the gas phase following high-energy collisions of ions of enantiopure amino acids with molecular chiral targets. Previously published experiments employed keV collisional activation under single-collision conditions (such conditions are not achievable in IMS) to test the possibility of chiral recognition under high-energy conditions [9]. However, no dependence on the chirality of the target gas was found in the relative intensities of the fragments formed in high-energy CID.

The present paper extends these studies to the low-energy regime: a collision energy of a few eV in the center-of-mass frame (CoM). Our objectives were first to detect formation of long-lived projectile-target complexes, and, second, to test if the efficiency of such gas-phase complexation reactions is dependent on the stereochemistry. Such experiments at precise CoM collision energies and under single-collision conditions could help in establishing a rational mechanism for the process of chiral separation in the gas phase when using a chiral target gas.

## Experimental

### Materials

l- and d-enantiomers of methionine (Met), phenylalanine (Phe), and tryptophan (Trp) with ≥98% optical purity were purchased from Sigma-Aldrich Sweden AB (Stockholm, Sweden) and used without further purification. (*R*)-2-butanol, (*S*)-2-butanol, a racemic mixture of (*R*)- and (*S*)-2-butanol enantiomers, and (*S*)-1-phenylethanol of 99% purity were obtained from Sigma-Aldrich and used without further purification as target gases. The amino acids were dissolved in a water/methanol/formic acid mixture (49:49:2 v/v/v) at a concentration of 0.04 mM.

### Methods

The experiments were carried out using the MS/MS mode of a hybrid quadrupole time of flight (Q-ToF) mass spectrometer (Q-ToF 2, Micromass, Manchester, UK) at the Department of Chemistry, Oslo University. Ions of the amino acids were produced by an electrospray ionization source in the positive mode of operation with 3 kV needle voltage and 100 °C source temperature. The precursor ions of the amino acid under study were isolated by the quadrupole analyzer, and then transferred to a hexapole collision cell. After reaction with the target gas, the ion products (and remaining reactants) were transferred to the ToF analyzer and detected with a microchannel plate-based detector. The target gas was introduced into the collision cell via a needle valve. The gas pressure in the collision cell was maintained in a range of 7.02–8.25 × 10^−4^ mbar during experiments with 2-butanol and 0.78–1.22 × 10^−4^ mbar with (*S*)-1-phenylethanol. The low pressure ensured single collision conditions for the ions traversing the collision cell, and that complex formation could be studied under well-defined conditions. The collision energies used in the measurements were 0.1–10 eV in the lab frame. Practically, these collision energies are being distributions due to the Doppler broadening (from the thermal motion of the collision gas) and spread in translational energy in the ion beam (we expect the former effect to be dominant in our case). At the lowest collision energies (0.1 eV in the lab frame), the translational energy in the CoM frame is equivalent to 300–400 K, i.e., the collisions can be considered thermal. At 4 eV lab frame collision energy, the broadening due to the Doppler effect (assuming 293 K for the gas) results in a distribution with a full-width-at-half-maximum of approximately 0.5 eV, which corresponds to 38%–50% of the nominal CoM collision energy; however, the average of the distribution is only 3% higher than the nominal collision energy [10, 11]. Nonetheless, since the presence of a distribution in the collision energies has little impact on the aim of this work, all reported energies are the nominal lab/CoM energies. When switching from one enantiomer of an amino acid to another, a procedure of electrospray-source cleaning was performed in-between by spraying a solution of water/methanol (50:50 v/v) through the system for several min, ensuring that all traces of the previous enantiomer was gone before introducing the new one. Product mass spectra were normally accumulated from 50 consecutive scans. For each measurement, the procedures of combining and smoothing were applied using the MassLynx v4.0 software. The relative abundance of each individual peak was obtained by normalizing to the total measured ion count and these data were used in the further data analysis.

## Results and Discussion

### Low-Energy Collisional Activation

It has been shown that high-energy collisional activation (1 keV CoM collision energy) of ions of enantiopure amino acids with chiral gas targets results in fragmentation of the projectile that is consistent with statistical energy distribution, and was seen to be free from stereochemistry-dependent effects [9]. In the current study, we focused on the low-energy collisional activation. The experiments were performed at single-collision conditions in the lab-frame energy range from 10 eV down to 100 meV. The results show that two processes occur at such conditions: (1) fragmentation of the projectile, and (2) formation of a projectile–target proton-bound complex.

### Fragmentation

**Figure 1.**
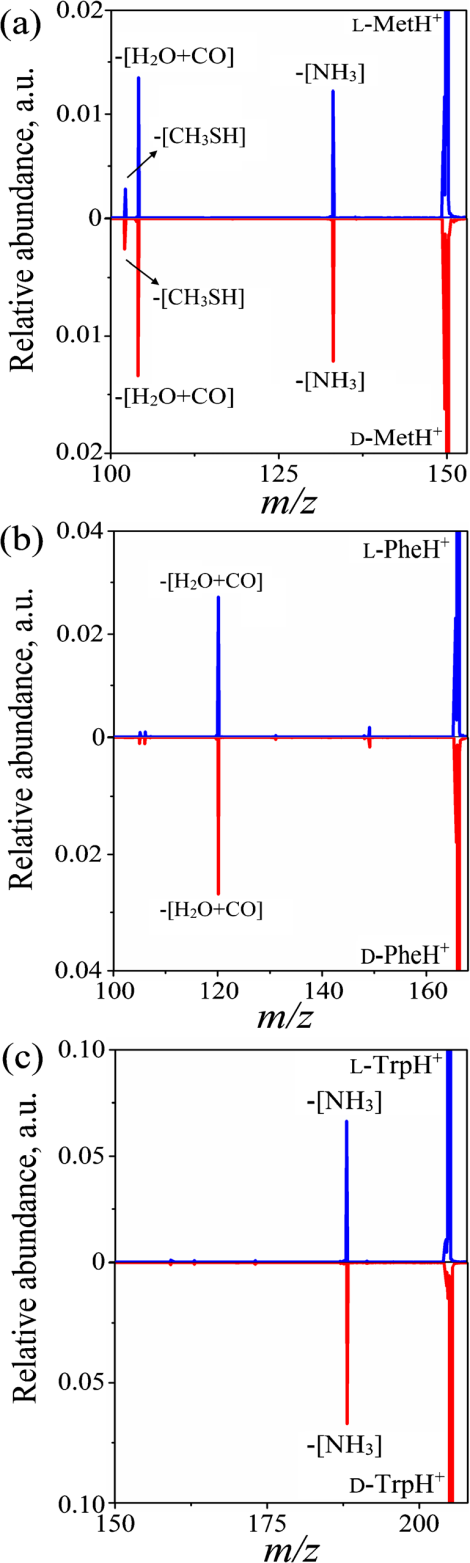
CID mass spectra obtained from collisions of **(a**) l- and d-Met, **(b)** l- and d-Phe, and **(c)** l- and d-Trp with (*S*)-2-butanol at the lab frame collision energy of 10 eV, which corresponds to collision energies of 3.3, 3.08, and 2.65 eV in the CoM frame. Mass spectra were normalized so that each parent ion has an abundance of 1

As could be expected, the product yield from low-energy CID was found to be much lower than in the case of high-energy collisions involving the same projectile ions and target [9]. The CID spectra obtained from fragmentation of protonated l-Met, Phe, and Trp after interacting with (*S*)-2-butanol at the lab frame collision energy of 10 eV are shown in Figure 1a–c.

Our results are consistent with the two accepted concepts used to explain the fragmentation of protonated ions of amino acids. The dissociation of the Phe moiety via the loss of H_2_O + CO, leading to the formation of the corresponding iminium ion at *m/z* = 120, can be described by the mobile proton model (Figure 1b) [9, 12, 13]. The dissociation of Trp by the loss of NH_3_ and consequent formation of a charged fragment with *m/z* = 188 is explained by the fragmentation model involving side chain group participation (Figure 1c) [9, 12, 13]. Both fragmentation channels take place in the course of Met dissociation leading to the formation of charged products with *m/z* = 104 (H_2_O + CO loss) and *m/z* = 133 (NH_3_ loss), see Figure 1a. The loss of CH_3_SH from the side chain group of Met giving *m/z* = 102 has also been observed (Figure 1a).

After careful data analysis, however, no influence of the stereochemical configuration of the projectile ions and the target gases on the fragmentation pattern and yield can be observed. As in the keV collision experiments reported above, the fragmentation spectra of the l- and d-enantiomers of Trp, Phe, and Met after 10 eV collisions (lab energy) with (*S*)-2-butanol target were found to be identical (Figure 1a–c). Utilization of a racemic mixture of 2-butanol enantiomers as a collision gas yielded the same relative fragment ion abundances (see Supplementary Figure S1 in Supporting Information). This indicates a mechanism for efficient transfer of the collision energy into internal degrees of freedom of the projectile, with further rapid energy randomization leading to indiscriminate fragmentation of the various enantiomeric pairs that only differ by small amounts of potential energy. Collisional energy uptake is likely to be due to impulsive collisions [14]; however, it cannot be determined precisely from our experiments.

### Complex Formation

**Figure 2.**
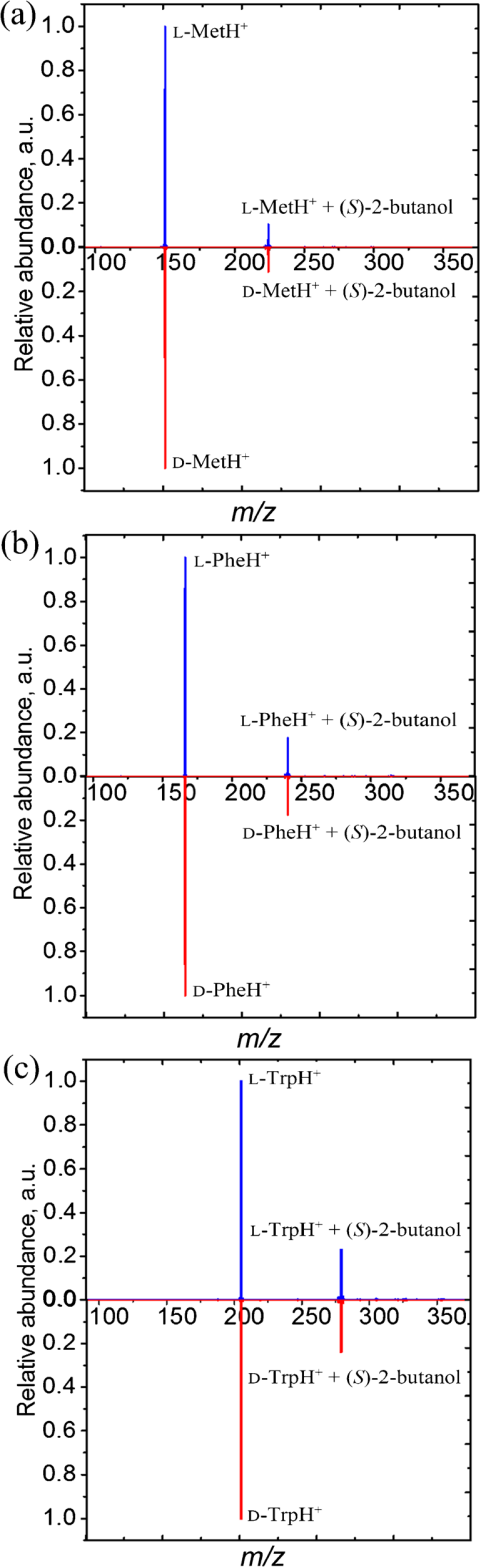
Mass spectra obtained from collisions of **(a)** l- and d-Met, **(b)** l- and d-Phe, and **(c)** l- and d-Trp with (*S*)-2-butanol at the lab frame collision energies of 0.1 eV, which corresponds to collision energies of 0.033, 0.030, and 0.027 eV in the CoM frame

All forms of the protonated amino acids were found to interact with the 2-butanol gas target by ion–molecule reactions to yield gas-phase complexes at very low collision energy. Figure 2a–c shows the mass spectra obtained when l- and d-forms of Met, Phe, and Trp were reacted with (*S*)-2-butanol at the lab frame collision energy of 0.1 eV. Each spectrum clearly shows a peak with a position that is shifted + 74 *m/z* units higher than the parent ion, which corresponds to the formation of a complex between the parent ion and the butanol target. The ion complex abundances were found to be indistinguishable for l- and d-amino acids after collisions with (*S*)-2-butanol (Figure 2a–c). Similarly, the efficiency of the complex formation reactions was observed to be identical for both forms of amino acids after collisions with (*R*)-form of 2-butanol (see Supplementary Figure S2 in Supporting Information).

**Figure 3.**
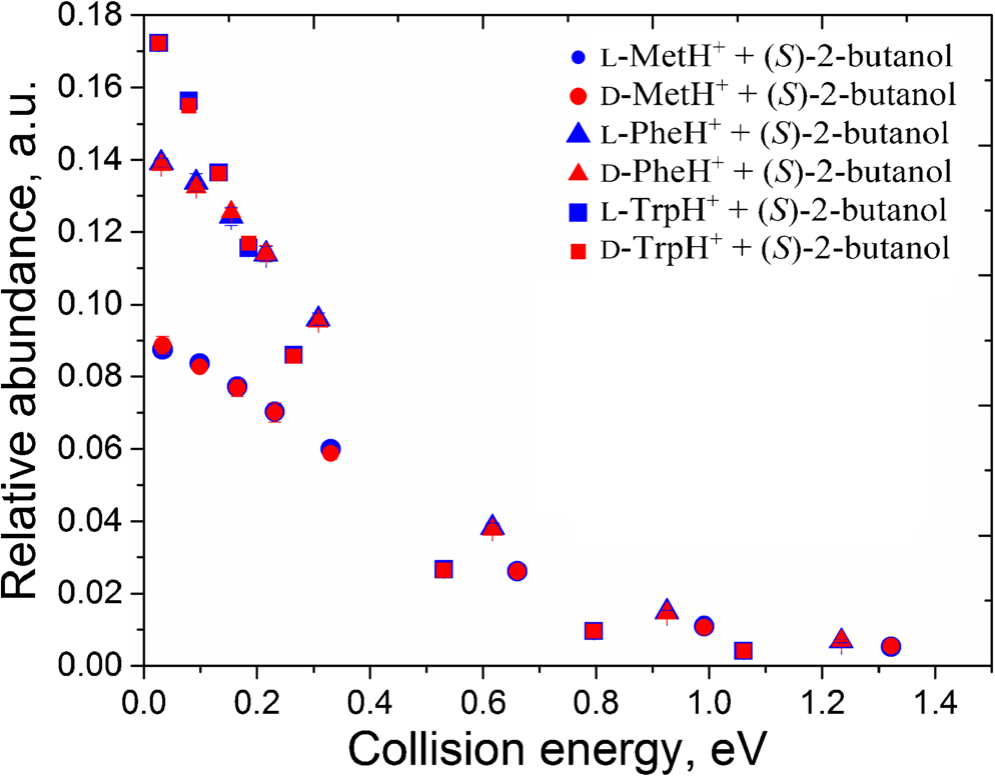
The abundance of proton-bound complexes of l- and d-Met, l- and d-Phe, l- and d-Trp with (*S*)-2-butanol generated in gas-phase collisions. Data is shown as a function of CoM collision energy

We found the above-mentioned observations intriguing as enantiopure (*R*)- and (*S*)-2-butanol have previously been used as drift-gas dopant in chiral IMS, and demonstrated to assist in distinguishing between enantiomers of various classes [5, 6]. The enantiomeric forms of the amino acids Met, Phe, and Trp were among those that could be distinguished in this manner [6]. To test further if chiral discrimination occurs already at the stage of the complex forming reaction, we carefully studied the efficiency of the complex formation as a function of the CoM collision energy. The range of the lab frame collision energies investigated was from 0.1 to 4 eV, and the data obtained for these reactions are plotted in Figure 3.

From Figure 3, it is apparent that the efficiency of the complex formation is independent of the stereochemical configuration of the reacting components. The abundances of the generated proton-bound complexes of l- and d-amino acids with (*S*)-2-butanol mirrored each other over the entire energy range. The results were found to be identical for collisions with (*R*)-2-butanol (these plots can be found in Supporting Information in Supplementary Figure S3).

In a publication describing the successful separation of enantiomers by modifying the drift gas with chiral 2-butanol in IMS, the mechanism behind the phenomenon was not established [6]. However, the authors suggested that the chiral recognition mechanism was likely based on the three-point interaction model: each enantiomer interacted with the modifier to a different extent, which changed the mobility of the analytes [6]. This mechanism of chiral recognition, also known as the Pirkle rule, is generally considered to be a prerequisite of any separation of enantiomers [15, 16].

However, an alternative mechanism of gas-phase chiral recognition has been recently suggested [7, 8]. Holness et al. [7] used achiral alcohols as drift gas dopants in high-resolution IMS to separate diastereomeric compounds, (*R,S*)-ephedrine and (*S,S*)-pseudoephedrine. These diastereomeric compounds could not be separated by IMS without doping the drift gas. The experimental results, also supported by theoretical calculations, revealed that the discriminating interactions between analytes and modifiers occur without the necessary condition of the modifier being a chiral molecule [7]. When an achiral volatile alcohol was injected into the drift tube, clusters were formed by multiple additions of the gas molecules to the analyte. Clustering induced conformational differences for (*R,S*)-ephedrine and (*S,S*)-pseudoephedrine, and led to the alteration of the cross-sectional area for each clustered diastereomer. As a result, each cluster migrated at a different mobility in the IMS drift tube [7].

For the amino acids investigated here, our results demonstrate that the efficiency of the ion–molecule complex formation is independent of the chirality of the projectile and target. Thus, the three-point interaction model is unlikely to be applicable in the case of chiral separations, which use 2-butanol as a chiral selector [6]. At the very least, chiral discrimination does not seem to happen during the first collision where the complex is initially formed. However, if we consider the multi-collisional techniques, such as IMS, our observations have some further implications. The complex formation reaction between the l- or d-amino acid and (*R*)- or (*S*)-2-butanol can be considered as the formation of a diastereomeric complex from gas-phase ions and chiral targets. Following this initial formation of the diastereomeric complex, further clustering, which is known to happen under IMS conditions, is likely to lead to the amplification of conformational differences, followed by the discrimination of the newly formed clusters according to the mechanism proposed by Holness et al. [7].

The concept of selective alteration of analytes gas-phase mobilities based on the formation of ion–molecule clusters is now being thoroughly investigated [17–22]. Indeed, the results of a just-published study, focusing on chiral differentiation of amino acids by IMS-MS through binuclear copper bound tetrameric ions, suggest that the mechanism for chiral recognition in IMS might be significantly different from that in tandem MS/MS [23]. Specifically, it was suggested that the MS/MS-based chiral recognition might be primarily dependent on the energy difference of the diastereomeric complexes, whereas the IMS-based chiral discrimination might be primarily governed by the size and shape difference of the diastereomeric cluster ions [23]. The assumption mentioned above agrees well with current results and also with our recent report on enantioselective CID of proton-bound diastereomeric complexes of Trp and 2-butanol [24]. The homochiral proton-bound complex of Trp with 2-butanol was found to be less stable in low-energy CID than a heterochiral one [24]. The enantioselectivity in the dissociation process was well detectable in MS/MS experiments [24]. Intriguingly, our current results show that the efficiency of the gas-phase complex-forming reactions of the same enantiopure compounds is instead free from chiral effects (Figure 2c, Figure 3).

**Figure 4.**
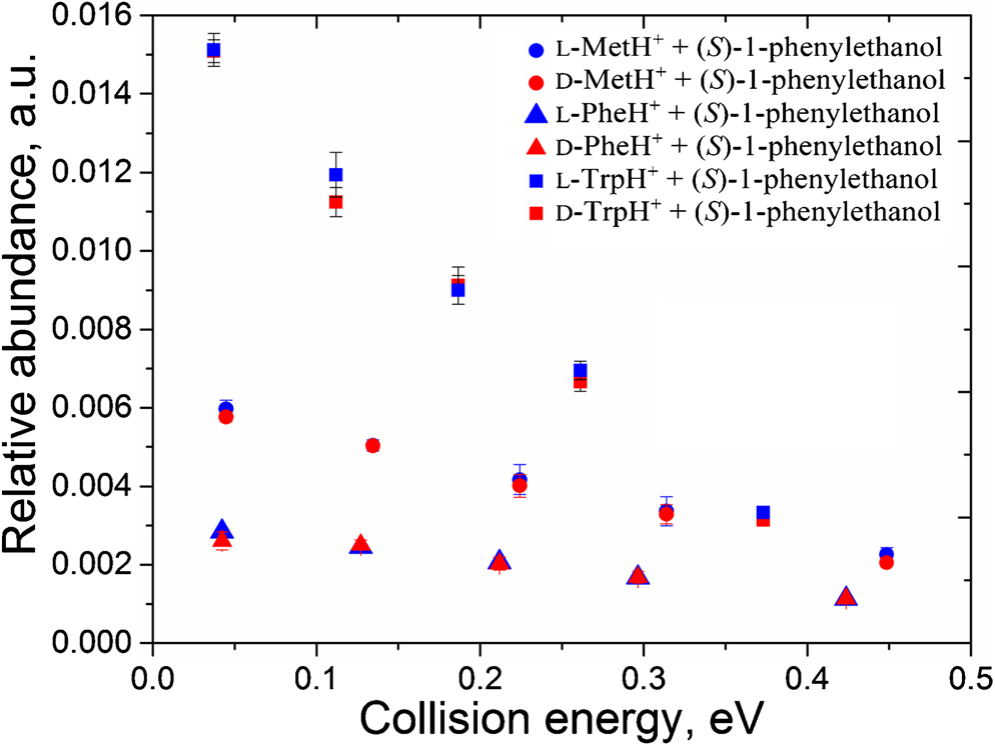
The abundance of proton-bound complexes of l- and d-Met, l- and d-Phe, and l- and d-Trp with (*S*)-1-phenylethanol generated in gas-phase collisions. Data is shown as a function of CoM collision energy

To better facilitate the steric interactions between analyte amino acid and a target gas molecule in the formed complexes, we used a bulkier target gas: (*S*)-1-phenylethanol. Collisions were performed in the range from 0.1 to 1 eV in the lab energy frame; both enantiomeric forms of Met, Phe, and Trp were investigated, and the results are plotted in Figure 4.

The experiments gave the same result as in the case of collisions with 2-butanol. The efficiency of complex-formation reactions between (*S*)-1-phenylethanol and the enantiopure amino acids was found to be independent of the chirality of the reacting species (Figure 4). This observation further supports the idea about the essential role of clustering in gas-phase chiral separations.

## Conclusions

In this study we investigated low-energy gas-phase collisions between protonated enantiopure amino acids (l- and d-forms of Met, Phe, Trp) and different chiral gas targets [(*R*)- and (*S*)-2-butanol, and (*S*)-1-phenylethanol]. Two processes have been found to occur over the studied meV–eV energy regime of collisional activation: fragmentation of the projectile ions and formation of proton-bound amino acid/target gas complexes. The efficiency of these processes, fragmentation and complex formation, have been found to be independent of the stereochemical configuration of the colliding partners. The fact that three-point interaction-based discrimination has not been observed in our experiments sheds light on a currently debated underlying concept of gas-phase chiral separations, and indirectly supports a new mechanism proposed by Holness et al. to describe the chiral selectivity that has been observed in chiral IMS measurements [7].

## Electronic supplementary material

ESM 1 (DOCX 686 kb)
