# Supplementary material for: Low-Energy Collisions of Protonated Enantiopure Amino Acids with Chiral Target Gases
Source: J Am Soc Mass Spectrom. 2017 Sep 21;28(12):2686–91. doi: 10.1007/s13361-017-1796-7 (PMC5707228; doi:10.1007/s13361-017-1796-7)
Supplement: Supplementary file 1 — (DOCX 686 kb) [file 13361_2017_1796_MOESM1_ESM.docx]

**Supporting Information**

**Low-energy collisions of protonated enantiopure amino acids with chiral target gases**

K. Kulyk,^1,2^ O. Rebrov,^1^ M. Ryding,^3^ R.D. Thomas,^1^ E. Uggerud,^3^ M. Larsson^1^

*^1^ Stockholm University, Department of Physics, SE-10691 Stockholm, Sweden*

*^2^ SCA R&D Centre, Sidsjövägen 2, SE-85121 Sundsvall, Sweden*

*^3^ University of Oslo, Department of Chemistry, NO-0315 Oslo, Norway*

Table of Contents

**Figure S1S3**

**Figure S2 S4**

**Figure S3 S5**


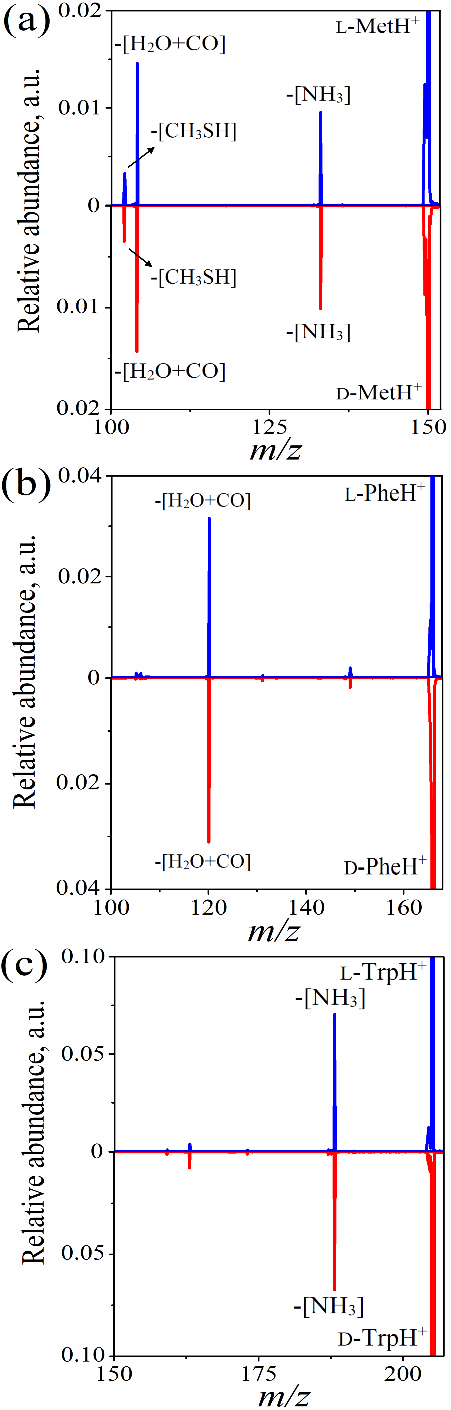


**Figure S1.** CID mass spectra obtained from collisions of (a) l- and d-Met, (b) l- and d-Phe, and (c) l- and d-Trp with racemic (*RS*)-2-butanol at the lab frame collision energy of 12 eV, which corresponds to collision energies of 3.97, 3.70, and 3.18 eV in the CoM frame. Mass spectra were normalized so that each parent ion has an abundance of 1.


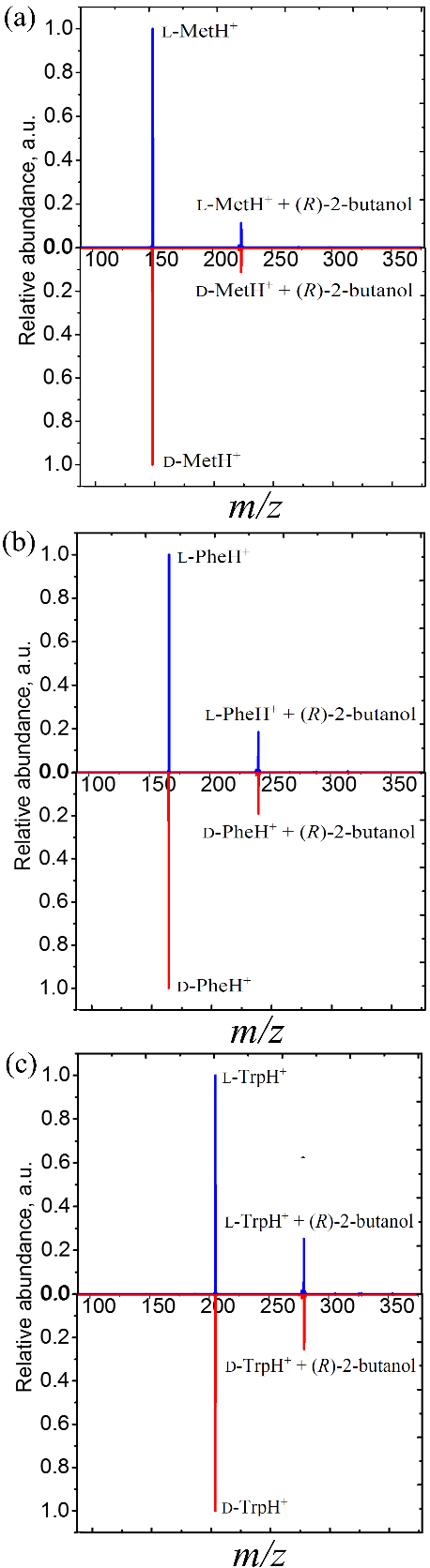


**Figure S2.** Mass spectra obtained from collisions of (a) l- and d-Met, (b) l- and d-Phe, and (c) l- and d-Trp with (*R*)-2-butanol at the lab frame collision energies of 0.1 eV, which corresponds to collision energies of 0.033, 0.030, and 0.027 eV in the CoM frame.


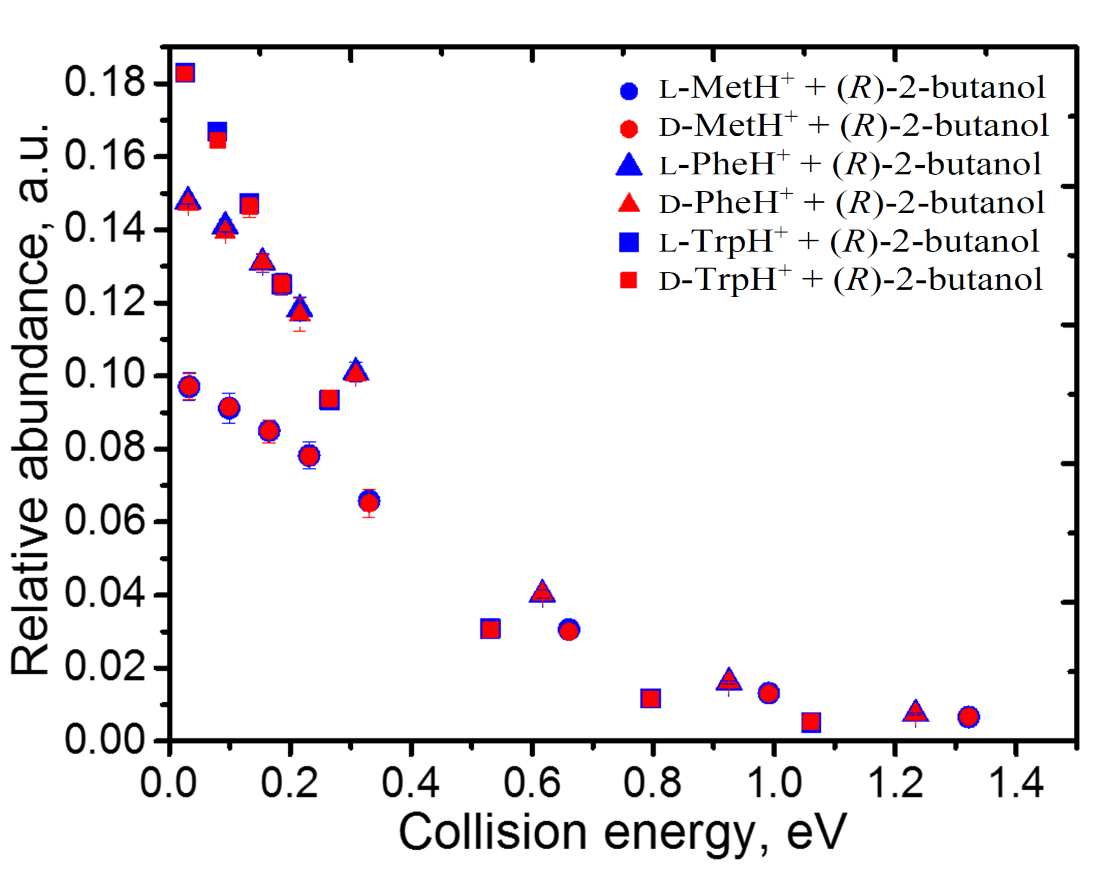


**Figure S3**. The abundance of proton-bound complexes of l- and d-Met, l- and d-Phe, l- and d-Trp with (*R*)-2-butanol generated in gas-phase collisions. Data is shown as a function of CoM collision energy.
